# Aberration-robust monocular passive depth sensing using a meta-imaging camera

**DOI:** 10.1038/s41377-024-01609-9

**Published:** 2024-09-05

**Authors:** Zhexuan Cao, Ning Li, Laiyu Zhu, Jiamin Wu, Qionghai Dai, Hui Qiao

**Affiliations:** 1 Department of Automation, Tsinghua University, Beijing, 100084 China; 2 Institute for Brain and Cognitive Sciences, Tsinghua University, Beijing, 100084 China; 3 Beijing National Research Center for Information Science and Technology, Tsinghua University, Beijing, 100084 China

**Keywords:** Imaging and sensing, Adaptive optics

## Abstract

Depth sensing plays a crucial role in various applications, including robotics, augmented reality, and autonomous driving. Monocular passive depth sensing techniques have come into their own for the cost-effectiveness and compact design, offering an alternative to the expensive and bulky active depth sensors and stereo vision systems. While the light-field camera can address the defocus ambiguity inherent in 2D cameras and achieve unambiguous depth perception, it compromises the spatial resolution and usually struggles with the effect of optical aberration. In contrast, our previously proposed meta-imaging sensor^1^ has overcome such hurdles by reconciling the spatial-angular resolution trade-off and achieving the multi-site aberration correction for high-resolution imaging. Here, we present a compact meta-imaging camera and an analytical framework for the quantification of monocular depth sensing precision by calculating the Cramér–Rao lower bound of depth estimation. Quantitative evaluations reveal that the meta-imaging camera exhibits not only higher precision over a broader depth range than the light-field camera but also superior robustness against changes in signal-background ratio. Moreover, both the simulation and experimental results demonstrate that the meta-imaging camera maintains the capability of providing precise depth information even in the presence of aberrations. Showing the promising compatibility with other point-spread-function engineering methods, we anticipate that the meta-imaging camera may facilitate the advancement of monocular passive depth sensing in various applications.

## Introduction

Depth sensing has become a cornerstone of various applications, such as robotics^2,3^, autonomous driving^4,5^, and augmented reality^6–8^. In these fields, accurate scene depth information is crucial, either as direct input or as a supervisory label for downstream tasks like scene understanding^9^, pose estimation^10^, and robotic manipulation^3^.

Due to the difficulty in optically encoding 3D information in a 2D image^11,12^, a common solution is to utilize active sensors such as Light Detection And Ranging^13^, time-of-flight camera^14^, or structured light camera^15^ to directly obtain precise scene depth through controlled illumination. However, their demand for controlled illumination results in high power consumption and low spatial resolution, making them expensive, bulky, and insensitive to scene detail. An alternative is to employ multiple 2D cameras to construct a multi-view-stereo vision system and calculate depth from the disparity between cameras^16^. Similarly, structure-from-motion leverages the motion of a single camera over time, matching pixels across different frames to triangulate the 3D position of matched pixels^17^. However, the precision of stereo vision systems is directly linked to the baseline between cameras or views, resulting in bulkiness and a reduced field of view for high-precision stereo vision systems^18^.

In contrast to the active sensors and stereo vision systems, obtaining depth directly from depth cues in monocular images offers a cost-efficient and compact alternative. Beyond utilizing relative depth cues like perspective, partial occlusions, or learned object shapes^19,20^, leveraging absolute depth cues contained in cameras’ point spread functions (PSFs) provides more physical constraints for accurate depth sensing and improved generalization across different scenes^21–23^. Conventional 2-dimensional cameras (2D) typically utilize defocus circles as absolute depth cues^23^. However, the defocus can significantly blur the image, and its symmetry around the focal plane introduces additional ambiguity in absolute depth sensing^21,22,24^. Traditional light-field cameras^25^ (LF) employ a microlens array (MLA) positioned between the sensor and the main lens, which helps them encode depth information through the disparity between different sub-aperture images. Although estimating depth from disparity is more straightforward than from defocus circles, the inherent low spatial resolution of each sub-aperture image diminishes the precision of disparity estimation and perception of overall scene detail. In contrast, our previously proposed meta-imaging sensor (Meta), built on the light-field camera design, incorporates an additional scanning mechanism. This innovation allows the meta-imaging camera to overcome the trade-off between spatial and angular resolution and achieve multi-site aberration correction through digital adaptive optics techniques (DAO), demonstrating its potential for achieving high-resolution imaging^1^.

In this work, we present a compact meta-imaging camera for monocular depth sensing that integrates the main lens, MLA, CMOS sensor, and piezo stage. Additionally, we propose an analytical framework for quantified precision analysis of monocular depth sensing across different cameras. Leveraging PSF models, we compute the Cramér–Rao lower bound (CRLB)^26^ for depth estimation, serving as theoretical precision of monocular depth sensing. Our analysis reveals that the meta-imaging camera exhibits higher precision over a broader depth range compared to light-field cameras, while also maintaining robust performance across varying signal-background ratios (SBR).

Moreover, our simulation reveals that the meta-imaging camera can effectively mitigate errors arising from unknown optical aberrations through DAO. To validate these findings, we implement an experimental system featuring both a 2D camera and a meta-imaging camera with identical optical parameters for the main lens and sensors. Combined with straightforward PSF-based depth estimation methods, our experiments further demonstrate that the meta-imaging camera can achieve accurate depth sensing even in the presence of aberration. Additionally, we indicate that promising compatibility between the meta-imaging camera and current PSF engineering techniques may facilitate the advancement of monocular passive depth sensing in various applications.

## Results

### Meta-imaging camera construction and PSF model

**Fig. 1:**
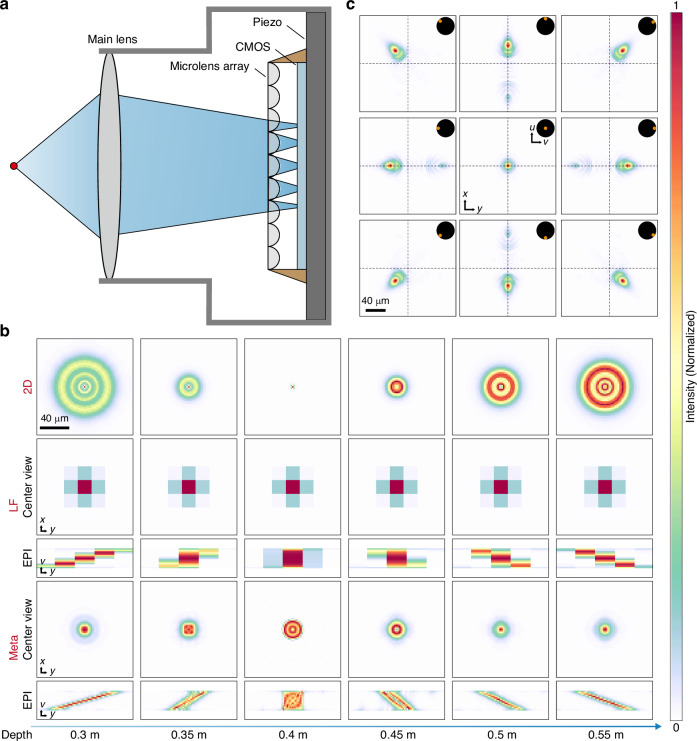
**The compact meta-imaging camera and PSF model based on wave optics**. An f/6 25 mm focus length main lens and an MLA with f/6, 27.75 μm pitch size, and 15 × 15 angle resolution are used to simulate the PSF. The focus distance is 0.4 m. **a** The meta-imaging camera integrates MLA, CMOS sensor, and piezo stage together. **b** Different views of the meta-imaging camera’s 4D PSF. **c** PSFs of different cameras at varying distances. The light-field camera and the meta-imaging camera illustrate the center view (8,8) and epipolar plane image (EPI) of 4D PSF

Our meta-imaging camera consists of a main lens, an MLA, a CMOS sensor, and a piezo stage. The MLA is fixed at a distance of one focal length from the CMOS sensor. The MLA and the CMOS sensor are fixed on the piezo stage, which enables accurate periodic scanning at high speed (Fig. 1a). To quantitively analyze the depth sensing performance of different monocular cameras and accurately estimate depth from the monocular image, it is essential to derive the PSF of different cameras, which involves modeling the propagation of light waves from an arbitrary 3D point through the optical system to the sensor measurements.

For the sake of simplicity, we consider the main lens as an ideal single thin lens. Assuming a point source located at the optic axis, the light wave first propagates to the front of the main lens as a spherical wave and passes through the main lens. Then the complex field Fresnel propagates to the front of the MLA, passes through MLA and finally Fresnel propagates to the sensor. Finally, the PSF of the 2D camera can be derived from the complex field before the MLA. The PSF of the light-field camera and the meta-imaging camera can be derived from the complex field in front of the sensor. The detailed propagation formula can be found in the Supplementary Note. S1.

As illustrated in Fig. 1b, both the light-field camera and the meta-imaging camera exhibit a larger depth of field compared to the 2D camera, indicating enhanced energy concentration away from the focal plane. However, compared to the meta-imaging camera, the spatial resolution of the light-field camera significantly decreased due to the inherent trade-off between spatial and angular resolution. Moreover, as depicted in Fig. 1b, c, for the meta-imaging camera, change in depth not only induces the disparity between different views but also causes alterations in diffraction patterns within each view. Additionally, applying geometric optics principles, the baseline between views in the meta-imaging camera is directly proportional to the aperture of the main lens^27^ (see Supplementary Note. S2 for the detailed formula). Hence, we can obtain two types of disparity, one is approximated by geometry optics, and the other is calculated through correlation operation between views of simulated PSF. As shown in Supplementary Fig. S1, our simulation PSF model based on wave optics and geometry optics model are compatible in terms of disparity.

### An analytical framework for the quantification of depth sensing precision

Monocular depth sensing can be deemed as the parameter estimation from the sample data, where depth serves as the distribution parameter and raw images serve as the sample data. The distribution varies depending on the camera and the scene. The mean squared error (MSE) of a parameter’s estimator can be decomposed into the variance and bias of the estimator. The minimum standard deviation of the parameter’s unbiased estimators is given by the square root of CRLB. The CRLB is given by the diagonal element corresponding to the parameter of the inverse of the Fisher information matrix. For a parameter vector $\theta =({\theta }_{1},{\theta }_{2},\cdots ,{\theta }_{N})$, an estimator vector $\hat{\theta }=({\hat{\theta }}_{1},{\hat{\theta }}_{2},\cdots ,{\hat{\theta }}_{N})$, a Fisher information matrix $I(\theta )\in {M}^{N\times N}$, and a likelihood function $f(x,\theta )$, the CRLB can be mathematically expressed as^26^

1 $${Var}(\hat{{\theta }_{i}})\ge {\left[{I}^{-1}\left(\theta \right)\right]}_{{ii}}\ge {\left[I(\theta )\right]}_{{ii}}^{-1},\,i=1,2,\cdots ,N$$

2 $$I\left(\theta \right)=E\left[{\left(\frac{\partial }{\partial \theta }{ln}\,f\left(x,\theta \right)\right)}^{\!\!T}\left(\frac{\partial }{\partial \theta }{ln}\,f\left(x,\theta \right)\right)\right],\,\theta \in \varTheta$$

Assuming the pixel discretization and Poisson noise model, we denote the mean value of each pixel as ${\nu }_{\theta ,k}$. According to the Poisson probability distribution function, the Fisher information matrix of a K-pixel image can be written as^28^

3 $$I\left(\theta \right)=\mathop{\sum }\limits_{k=1}^{K}{\left(\frac{\partial {\nu }_{\theta ,k}}{\partial \theta }\right)}^{T}\left(\frac{\partial {\nu }_{\theta ,k}}{\partial \theta }\right)\frac{1}{{\nu }_{\theta ,k}}$$

Note that ${\nu }_{\theta ,k}$ can be further decomposed into a component from the target ${\mu }_{\theta ,k}$, and a component from the background ${\beta }_{k}$. Typically, the parameter of interest, such as the target’s depth, will affect ${\mu }_{\theta ,k}$, while ${\beta }_{k}$ remains constant. Using the decomposition, the Fisher information matrix can be rewritten as

4 $$I\left(\theta \right)=\mathop{\sum }\limits_{k=1}^{K}{\left(\frac{\partial {\mu }_{\theta ,k}}{\partial \theta }\right)}^{T}\left(\frac{\partial {\mu }_{\theta ,k}}{\partial \theta }\right)\frac{1}{{\mu }_{\theta ,k}+{\beta }_{k}}$$

The K-pixel images are generated by the convolution between the PSF of the camera and the target. We can take an ideal point source with a constant background as the target and the target’s depth as parameter $\theta$ to calculate the CRLB of different monocular cameras, which can be deemed as their theoretical depth sensing precision physically constrained by cameras and scenes.

### Depth sensing performance comparison among different monocular cameras

For quantitative analysis comparing the depth sensing precision of different monocular cameras, we use an optical setup comprising an f/6 25 mm focus length main lens, an MLA with f/6, 14.8 μm pitch size, and 8 × 8 angle resolution.

**Fig. 2:**
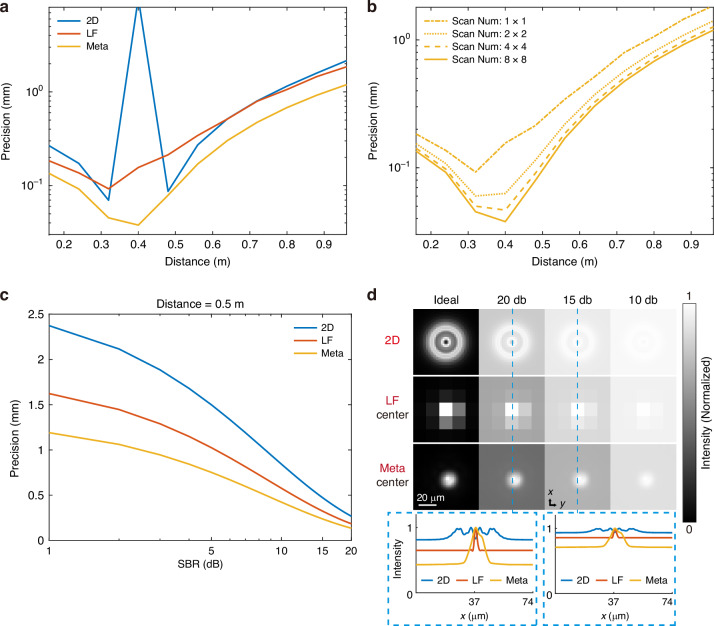
**Comparative analysis of depth sensing precision among different cameras.** The meta-imaging camera employs a full scan (8 × 8) by default and the focus distance is 0.4 m. The signal-background ratio is calculated through the ratio of the targets’ total photon number and background photon number. **a** Depth sensing precision at various distances for all three cameras with SBR set at 20 dB. **b** Depth sensing precision at various distances for the meta-imaging camera with different scan numbers. **c** The curves of depth sensing precision versus different SBR for different cameras. **d** Simulated point source images of different cameras with varying SBR. The distance of the point source is fixed at 0.5 m

As shown in Fig. 2a, the depth sensing precision of the 2D camera significantly decreases near the focal plane. This phenomenon arises from the nearly invariant PSF of the 2D camera within the depth of field, leading to a loss of depth information near the focal plane. While the light-field camera mitigates this limitation by capturing additional angle information, its precision diminishes at certain distances due to a reduction in spatial resolution. In contrast, the meta-imaging camera not only precisely estimates the depth of targets near the focal plane but also exhibits superior precision at other distances. This is attributed to the meta-imaging camera’s unique capability to acquire angle information without compromising spatial resolution, thereby retaining more depth information about the scene. Figure 2b and Supplementary Fig. S2 illustrate that the precision of depth sensing improves with the scan number owing to the enhancement in spatial resolution. Notably, increasing the scan number from 1 (i.e., light-field cameras) to 2 (i.e., meta-imaging cameras scanning 2 × 2 times within a microlens area) yields significant improvements at a relatively low cost, indicating that a lower scan number such as 2 or 3 is a more cost-effective choice in practical scenarios.

Figure 2c reveals that depth sensing precision increases with SBR for all three cameras. The SBR is calculated through the ratio of the targets’ total photon number and background photon number. However, when SBR decreases, the depth sensing precision of the 2D camera declines more rapidly than that of the light-field camera and the meta-imaging camera. This discrepancy is attributed to the microlens array presented in them, which encodes changes in 2D cameras’ defocus circle into relative shifts between different sub-apertures and alterations of the diffraction pattern within each sub-aperture. Crucially, the diffraction pattern within each sub-aperture changes more gradually with depth compared to the defocus circle of the main lens. Consequently, the light-field camera and the meta-imaging camera exhibit better energy concentration at distances away from the focal plane (Fig. 2d), offering enhanced robustness to SBR compared to the 2D camera. Due to its higher spatial resolution, the meta-imaging camera demonstrates even greater resistance under low SBR conditions compared to the light-field camera (Fig. 2c). Additionally, the meta-imaging camera is more robust to focus distance variance compared to the 2D camera as well (Supplementary Fig. S3).

### Aberration-present depth sensing experiments

The imaging systems in practical scenarios are often affected by optical aberration arising from imperfections in system design and imaging conditions. Optical aberration not only deteriorates image quality but also downgrades depth sensing performance. To explore the effect of aberration on depth sensing, we simulated point source images distorted by aberrations, estimated depth using maximum likelihood estimation (MLE) without prior knowledge of the underlying aberrations, and calculated the root mean square error (RMSE) of predicted depth.

**Fig. 3:**
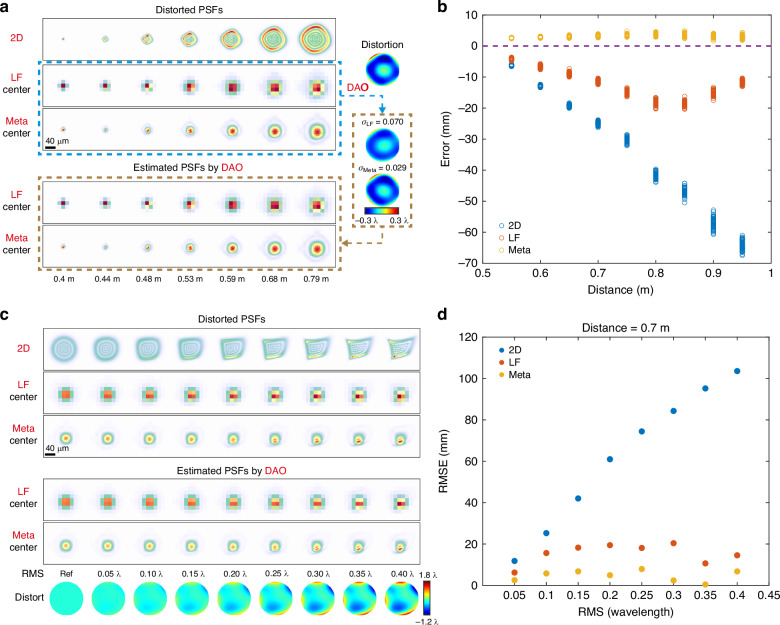
**Impact of optical aberration on depth sensing performance**. We simulated 50 images of a point source at each distance. The focus distance is 0.4 m. σ is calculated through the ratio of the residual phase root mean square to the peak-to-valley value of the added aberration, lower indicates better aberration estimation. RMSE is the root mean square error between depth predictions and ground truth. RMS is the root mean square of the aberration. **a** PSFs distorted by aberration and PSF estimated through DAO techniques of the light-field camera and the meta-imaging camera. **b** Estimation error of different cameras. **c** PSFs distorted by the aberration with different levels and corresponding PSFs estimated through DAO. **d** The curve of RMSE versus RMS of the aberration. The distance of the point source is fixed at 0.7 m (**c**, **d**)

As shown in Fig. 3, optical aberrations distort normal PSFs, thereby introducing significant errors in depth estimation due to the discrepancy between ideal PSFs used for estimation and actual PSFs distorted by aberrations. In this context, the light-field camera and the meta-imaging camera exhibit a notable advantage. Through DAO, they can estimate optical aberration from relative shifts between different sub-aperture images. Leveraging the estimated aberration, they can obtain a PSF model closer to the distorted PSFs (Fig. 3a), resulting in significantly lower estimation bias compared to the 2D camera (Fig. 3b). Moreover, even with increased aberrations, the light-field camera and the meta-imaging camera maintain stable performance, whereas the RMSE of the 2D camera notably increases. Furthermore, the meta-imaging camera, benefiting from higher spatial resolution and thus enhanced accuracy in aberration estimation, demonstrates considerably lower estimation error compared to the light-field camera (Fig. 3b, d, Supplementary Fig. S4).

**Fig. 4:**
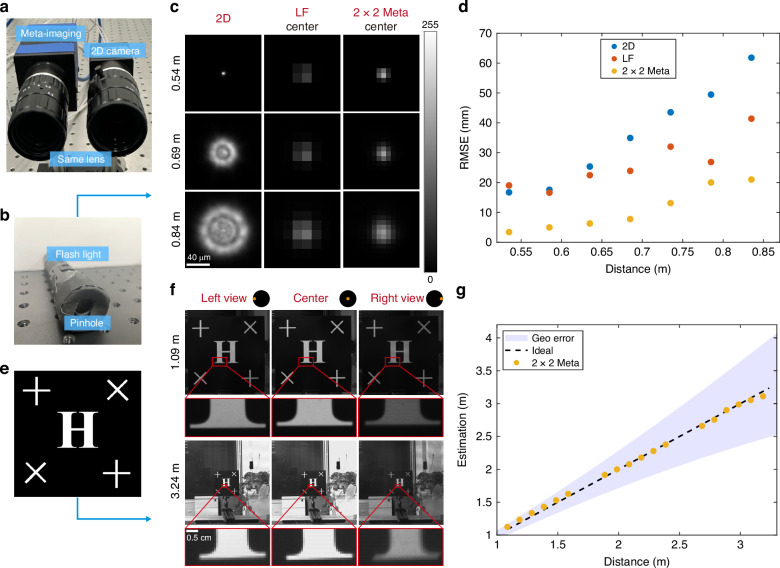
**Experimental depth sensing with different monocular cameras**. **a** A 2D camera and a meta-imaging camera, both possessing identical optical parameters. **b** The point source target consists of a flashlight and pinhole. **c** Experimental point source images at different distances of different cameras. Set the camera focus at 0.45 m. **d** Point depth estimation RMSE of different cameras. **e** A board with “H”. **f** Experimental board images at different distances of the meta-imaging camera. Set the meta-imaging camera focus at 2.48 m. **g** Estimated depth of the board using deconvolution and PSF model. The “geo error” refers to theoretical depth estimation error based on geometry optics

In our experiment, we deployed a 2D camera and a meta-imaging camera, both configured with optical parameters used in our simulations (a main lens with f/6 and 25 mm focus length, an MLA with f/6, 14.8 μm pitch size, and 8 × 8 angle resolution) (Fig. 4a). The main lenses contain slight system aberration. First, we set up a scene consisting of a flashlight equipped with a pinhole to approximate the point source target at various distances, as depicted in Fig. 4b. This setup enabled the acquisition of raw point source images for different cameras (Fig. 4c).

To mitigate the inherent defocus ambiguity of 2D cameras and compare the depth sensing performance of the 2D camera and the meta-imaging camera, we set the 2D camera and the meta-imaging camera focus at 0.45 m and captured point source images across a distance ranging from 0.54 m to 0.84 m. The depth was estimated through MLE and the simulation PSF model. The results, presented in Fig. 4d, indicate that the meta-imaging camera scanning 2 × 2 times within a microlens area (2 × 2 meta-imaging camera) exhibits significantly lower error compared to both the 2D camera and light-field camera. This superiority stems from the meta-imaging camera’s ability to obtain angular information with high spatial resolution. Moreover, the meta-imaging camera’s capacity to accurately correct unknown optical aberration minimizes the discrepancy between simulation and experimental PSFs, thereby decreasing the depth estimation bias. While the light-field camera captures angular information as well, its reduced spatial resolution compromises aberration estimation accuracy. As a result, its depth estimation performance marginally surpasses that of the 2D camera only at distances away from the focal plane. Additionally, the performance improvement gained by increasing the scan number from 2 to 8, as indicated by the reduction in mean RMSE from 13 mm to 9 mm, is less pronounced compared to the improvement achieved by replacing a light-field camera with a 2 × 2 meta-imaging camera, which reduces mean RMSE from 32 mm to 13 mm (Supplementary Fig. S5). This suggests that a lower scan number remains viable for practical applications (see Supplementary Fig. S9 and Supplementary Note. S4 for the detailed discussion).

Moreover, to valid the depth sensing performance of the meta-imaging camera in more practical scenarios, we set up a scene consisting of a board adorned with a character “H” and some “+” pattern, as shown in Fig. 4e. We captured the images of the board at different distances using the meta-imaging camera, spanning from 1 m to 3.5 m. Employing a naïve method based on deconvolution and PSF, we can obtain the board depth from a monocular board image. Although the disparity between views is small (Fig. 4f), we still achieve a mean absolute error of 0.03 m and a mean relative error of 1.7%.

According to the geometry optics conclusion, the maximum equivalent baseline between different views of the meta-imaging camera is the aperture size of the main lens^27^. The precision of a binocular stereo-vision system is given below. $z$ is depth, $B$ is the baseline, $f$ is the focus length of the camera, and $\Delta d$ is disparity estimation’s precision.

5 $$\Delta z=\frac{{z}^{2}}{{Bf}}\Delta d$$

As shown in Fig. 4g, our depth estimation error is significantly lower than the theoretical error given by Eq. (5). This improvement is attributed to our utilization of a PSF model based on wave optics, offering a more accurate description of the imaging process compared to geometric optics. Thus, our method extracts more depth information from the measurements, leveraging not only the disparity between different views but also the variation in diffraction patterns within each view to estimate depth.

### Depth sensing precision of cameras integrated with a double-helix phase mask

Due to limitations such as depth information loss within the depth of field and defocus ambiguity caused by PSF symmetry around the focal plane, the 2D camera often utilizes PSF engineering techniques like astigmatism^21,29,30^ and double-helix phase mask^31–33^ to mitigate ambiguity and maintain more depth information. To comprehensively compare three different monocular cameras, we design a double-helix phase mask for the 2D camera and apply it directly on the light-field camera and the meta-imaging camera in simulation. The double-helix phase mask, initialized with Fresnel zones^34^ and optimized through iterative Fourier transforms algorithm^32,35^, enables PSFs of the 2D camera configured with the same optical parameters as previous simulations to rotate 100° from 0.3 m to 0.8 m (see Supplementary Note. S3 for the detailed principle of phase mask design and Supplementary Fig. S6 for the comparison between initial PSFs and optimized PSFs).

**Fig. 5:**
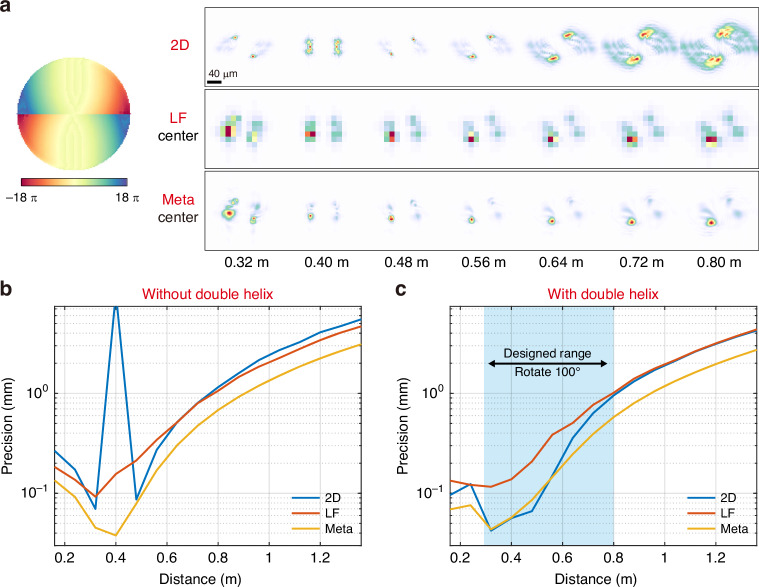
**Impact of double-helix PSF engineering on monocular depth sensing using different cameras**. Use an f/6 25 mm focus length main lens and an MLA with f/6, 14.8 μm pitch size, and 8 × 8 angle resolution to design a phase mask and simulate PSF. The focus distance is 0.4 m. The double helix phase mask enables the PSF of the 2D camera to rotate 100° from 0.3 m to 0.8 m. **a** The corresponding phase mask and simulation PSFs of three monocular cameras. **b** The curve of depth sensing precision of all three cameras without double helix phase mask versus depth. **c** The curve of depth sensing precision of all three cameras with double helix phase mask versus depth

As depicted in Fig. 5, integrating a double-helix phase mask significantly boosts the depth sensing precision of the 2D camera. However, for the light-field camera and the meta-imaging camera, the phase mask leads to a decrease in depth sensing precision near the focus plane, with improvements observed at other depths. Despite the superior precision of the 2D camera within the specific depth range for which the phase mask was designed, the meta-imaging camera consistently outperforms it across the entire depth range. These findings suggest that the meta-imaging camera retains compatibility with PSF engineering techniques, indicating the potential for future development of PSF engineering techniques tailored specifically for the meta-imaging camera.

## Discussion

The meta-imaging camera can not only achieve precise monocular depth sensing but also enhance the depth sensing capabilities of current stereo vision systems. CRLB of the binocular stereo vision system using meta-imaging cameras is much lower than that of the system using 2D cameras across the entire depth range. Moreover, meta-imaging cameras allow stereo vision systems to utilize large aperture lenses without compromising depth sensing precision at distances away from the focal plane (Supplementary Fig. S7). Therefore, the meta-imaging camera can effectively substitute the 2D camera in current stereo vision systems, enhancing their depth sensing performance and robustness, particularly in low-light and aberration-present conditions.

Variations in air temperature and pressure, referred to as atmospheric turbulence (AT), introduce random fluctuations to the refractive index of the medium, causing distortions in the wavefront. The impact of AT becomes particularly pronounced in long-distance imaging, typically exceeding 100 m. Consequently, the precision of existing long-distance monocular passive depth sensing methods is significantly compromised by unknown AT^33^. Additionally, in large baseline stereo vision systems, the performance of disparity estimation deteriorates substantially at long distances due to image distortion and blur caused by AT. Leveraging the aberration correction capability of the meta-imaging camera, we posit that it holds the potential to enable accurate and robust depth sensing in challenging long-distance scenarios for both monocular and multiocular imaging systems.

In summary, we have demonstrated the meta-imaging camera can capture more depth information from the scene compared to the light-field camera and the 2D camera. The meta-imaging camera exhibits enhanced robustness to changes in SBR. Moreover, the meta-imaging camera’s capability to correct optical aberration from captured measurements enables it to maintain precise depth sensing under imperfect imaging conditions. While the pre-calibration can assist the 2D camera in correcting static aberration originating from the optical system, it is ineffective against dynamic aberration arising from the scene itself. These advantages open up a wide array of potential applications for monocular passive depth sensing with meta-imaging cameras, particularly in fields such as augmented reality, autonomous driving, and robotics, where accurate and robust depth sensing is crucial.

## Methods

### MLE-based point depth estimation

We employ a MLE for the estimation of point source depth in both simulation and experimental settings. Our sensor model accounts for only Poisson noise. The parameters to be estimated include the point source location $(x,y,z)$, intensity $A$, and background intensity $B$. To convert the measurements to photon count, experimental cameras are calibrated to obtain gain and offset. MATLAB optimization toolbox is used for likelihood maximization represented below.

6 $$\left(x,y,z,A,B\right)={argmin}\left(\mathop{\sum }\limits_{k=1}^{K}{v}_{\theta ,k}\log \left({\mu }_{\theta ,k}+{\beta }_{\theta ,k}\right)-{\mu }_{\theta ,k}-{\beta }_{\theta ,k}\right)$$

Where $K$ is the total pixel number of measurements, ${v}_{\theta ,k}$ is the measurements following the Poisson distribution with a mean of ${\mu }_{\theta ,k}+{\beta }_{\theta ,k}$, ${\mu }_{\theta ,k}$ is determined by the PSF model and the location $(x,y,z)$, and ${\beta }_{\theta ,k}$ is determined by the PSF model and the background intensity $B$.

Because of the 2D camera’s ambiguity around the focal plane caused by the symmetry of defocus, we constrain the depth of the point source to be on one side of the camera’s focal plane only to ensure that there is no ambiguity when using a 2D camera for depth estimation. For the light-field camera and the meta-imaging camera, aberration is estimated from the point source images near the focal plane. The estimated aberration is subsequently incorporated into the simulation PSF model, which is then utilized in the optimization process of MLE.

### PSF-based depth estimation via deconvolution

To estimate the depth of the board, we first crop the region that exclusively contains the board from the raw image. Subsequently, we apply a reconstruction algorithm previously proposed^1^ to estimate optical aberration and recover the full spatial resolution latent image. The algorithm follows an iterative approach, where each iteration entails deconvolution using the current PSF, aberration estimation based on the deconvolution result, and generation of a new PSF incorporating the estimated aberration. This iterative process continues until convergence is achieved in the aberration estimation result.

Since the defocus term in estimated aberration represents the target’s distance from the focal plane, we can convert the focus PSF with defocus aberration to a PSF with a certain distance from the focal plane through our PSF model and a naïve optimization process. We take that distance as the coarse estimated depth and use the estimated aberration without defocus term and PSF model to generate an experimental 3D PSF around the coarse depth. Then, we convolve the experimental 3D PSF with the latent image to create a synthetic blurred image. The convolution error is then calculated between this synthetic image and the actual experimental image. The distance whose PSF has minimal convolution error is the final depth estimation result. As illustrated in Supplementary Fig. S8, the estimation error of refined depth is much smaller compared to that of coarse depth.

## Supplementary information

Supplementary Information

## Data Availability

The data that support the plots within this paper and other findings of this study are available from the corresponding authors upon reasonable request.

## Code availability

The code that supports the plots within this paper and other findings of this study is available from the corresponding author on request.
