# Supplementary material for: Aberration-robust monocular passive depth sensing using a meta-imaging camera
Source: Light Sci Appl. 2024 Sep 5;13:236. doi: 10.1038/s41377-024-01609-9 (PMC11377717; doi:10.1038/s41377-024-01609-9)
Supplement: Supplementary file 1 — Supplementary Information [file 41377_2024_1609_MOESM1_ESM.docx]

# Supplementary Information for Ab**erration-robust monocular passive depth sensing using a meta-imaging camera**

Zhexuan Cao^1,2,3^, Ning Li^1,2,3^, Laiyu Zhu^1,2,3^, Jiamin Wu^1,2,3^, Qionghai Dai^1,2,3*^ & Hui Qiao^1,2,3*^

^*^Correspondence:qhdai@tsinghua.edu.cn, qiaohui@mail.tsinghua.edu.cn

## Contents:

**Supplementary Fig. S1.** Disparity separately calculated from the PSF model and geometry optics model.

**Supplementary Fig. S2.** Impact of SBR and scan number on depth sensing precision.

**Supplementary Fig. S3.** Impact of focus distance on depth sensing precision of different monocular cameras.

**Supplementary Fig. S4.** Impact of optical aberration on depth sensing performance.

**Supplementary Fig. S5.** Point depth estimation RMSE of the light-field camera, 2x2 meta-imaging camera, and 8x8 meta-imaging camera.

**Supplementary Fig. S6.** Initial and optimized phase mask and PSFs.

**Supplementary Fig. S7.** Depth sensing precision of binocular stereo vision system using different cameras.

**Supplementary Fig. S8.** Estimation error of coarse depth and refined depth.

**Supplementary Fig. S9.** The curve of depth sensing precision of 2x2 meta-imaging cameras and light-field cameras with different pixel sizes.

**Supplementary Note.S1.** Detailed propagation formula

**Supplementary Note. S2.** Baseline of light-field cameras

**Supplementary Note. S3.** Principle of double-helix phase mask design

**Supplementary Note. S4.** The importance of scanning mechanism for depth sensing performance improvement

## Supplementary Figures


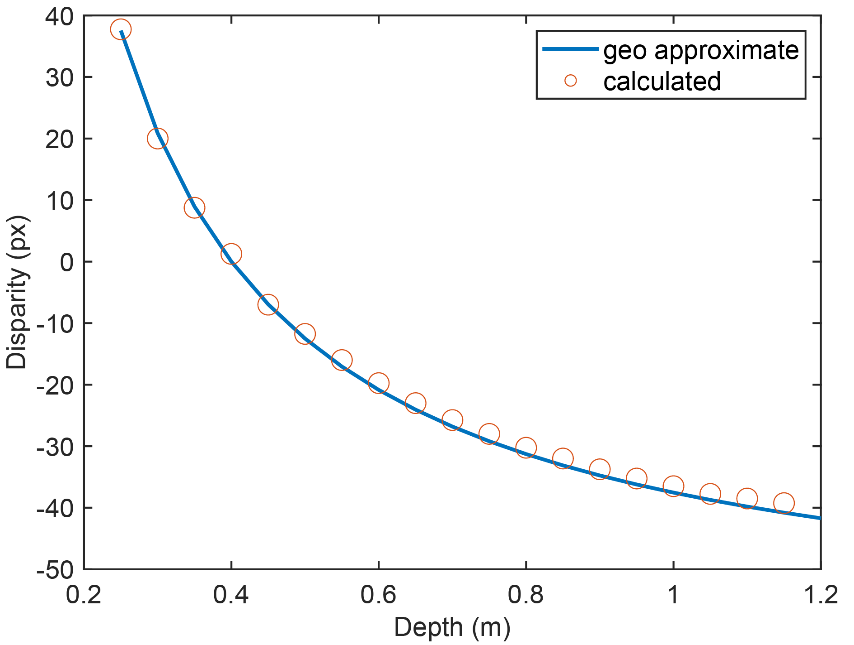
**Supplementary Fig. S1** **| Disparity separately calculated from the PSF model and geometry optics model.** The proposed PSF model based on wave optics and geometry optics model is compatible in terms of disparity.

**
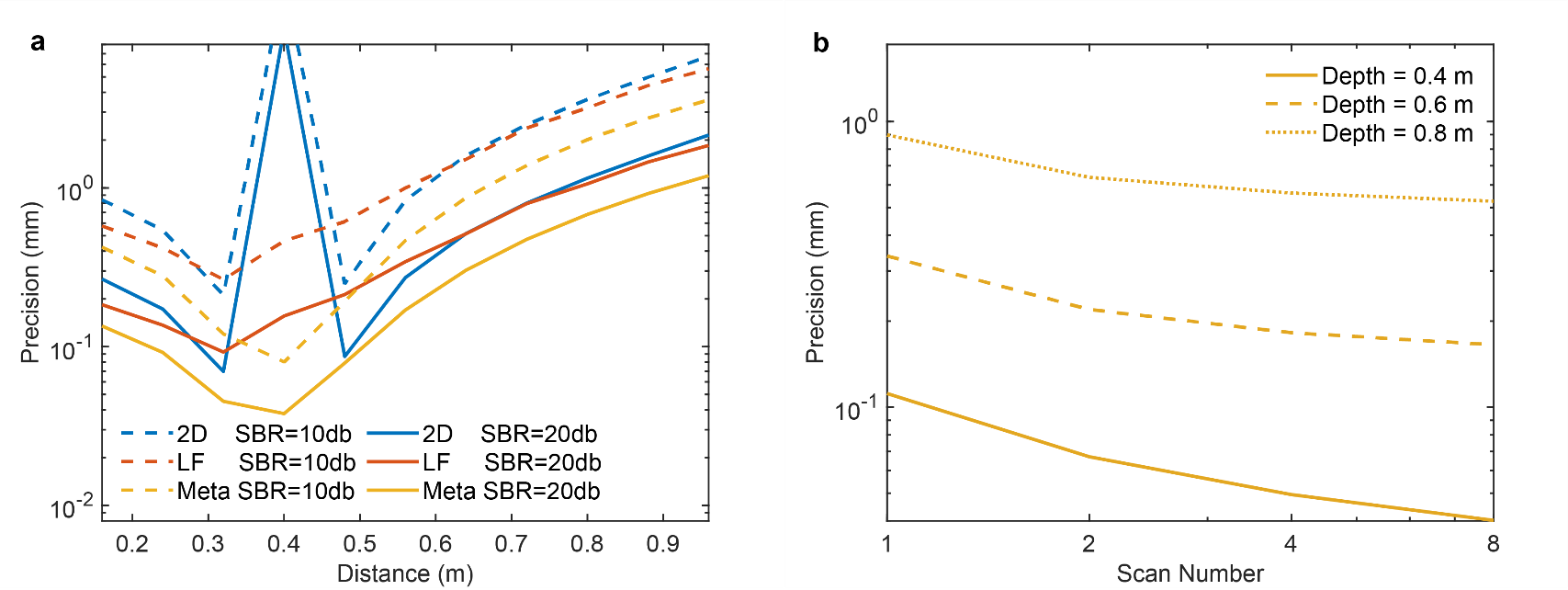
Supplementary Fig. S2** **| Impact of SBR and scan number on depth sensing precision**. **a.** The curve of three monocular cameras’ depth sensing precision versus distance with SBR set as 10 dB and 20 dB. Compared to the meta-imaging camera, 2D and light-field cameras’ precision decreases faster when SBR decreases. **b.** The curve of the meta-imaging camera’s depth sensing precision versus scan number. The biggest improvement is observed when increasing the scan number from 1 to 2.


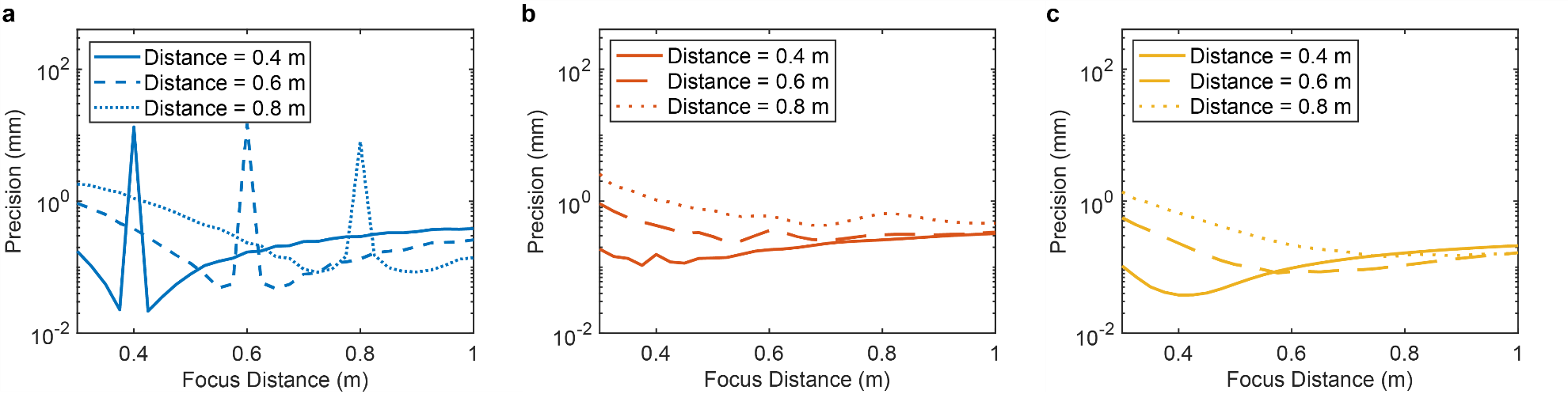
**Supplementary Fig. S3** **| Impact of focus distance on depth sensing precision of different monocular cameras. a.** The curves of depth sensing precision using the 2D camera versus different focus distances at different distances. **b.** The curves of depth sensing precision using the light-field camera versus different focus distances at different distances. **c.** The curves of depth sensing precision using the meta-imaging camera versus different focus distances at different distances.


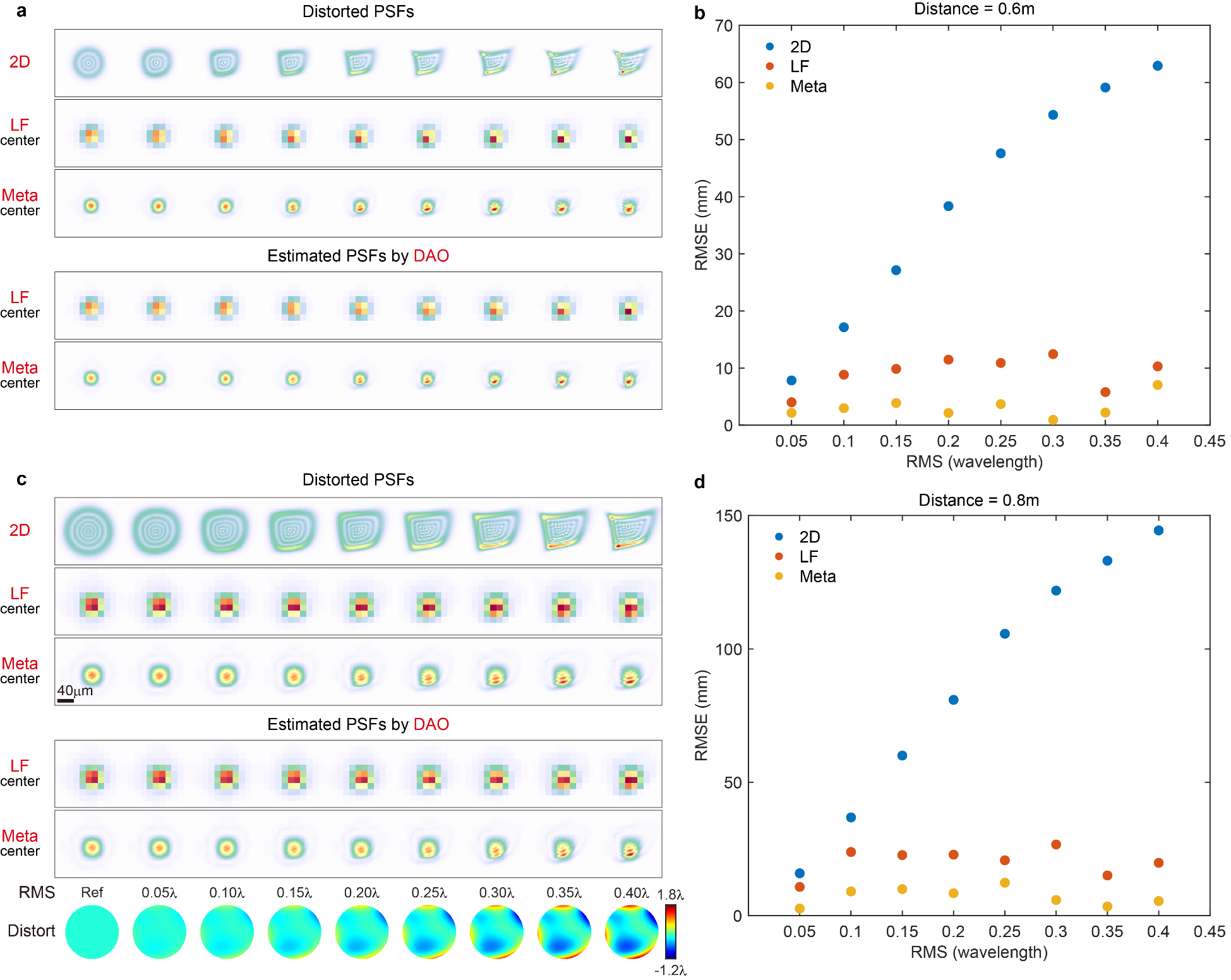
**Supplementary Fig. S4** | **Impact of optical aberration on depth sensing performance.** We simulated 50 images of a point source at each distance. The focus distance is 0.4m. RMSE is the root mean square error between depth predictions and ground truth. RMS is the root mean square of the aberration. **a.** PSFs distorted by the aberration with different levels and corresponding PSFs estimated through DAO. **b.** The curve of RMSE versus RMS of the aberration. The distance of the point source is fixed at 0.6m (**a,b**). **c.** PSFs distorted by the aberration with different levels and corresponding PSFs estimated through DAO. **d.** The curve of RMSE versus RMS of the aberration. The distance of the point source is fixed at 0.8m (**c,d**).


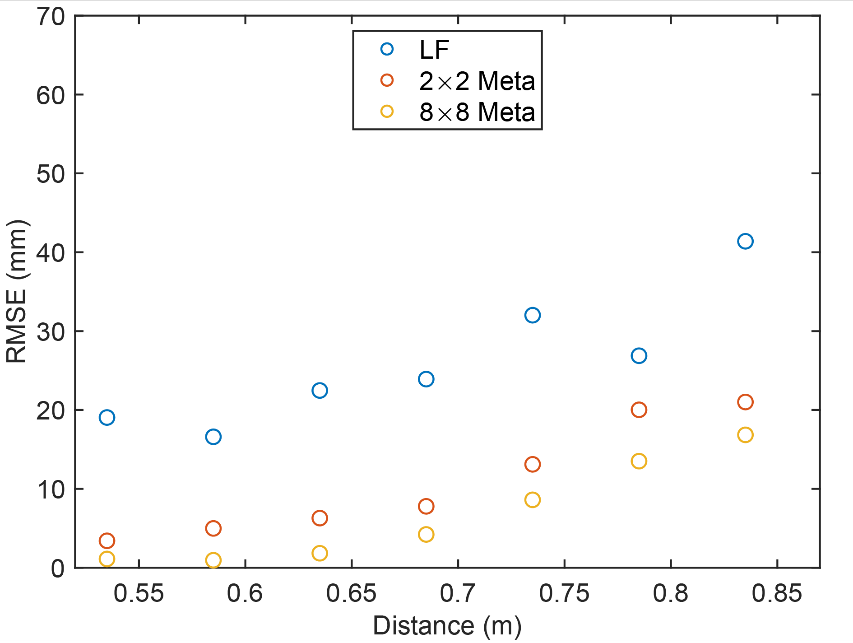
**Supplementary Fig. S5** **| Point depth estimation RMSE of the light-field camera, 2x2 meta-imaging camera, and 8x8 meta-imaging camera.** The performance improvement gained by increasing the scan number from 2 to 8, as indicated by the reduction in mean RMSE from 13mm to 9mm, is less pronounced compared to the improvement achieved by replacing a light field camera with a 2x2 meta-imaging camera, which reduces mean RMSE from 32mm to 13mm. This suggests that a lower scan number remains viable for practical applications.

**
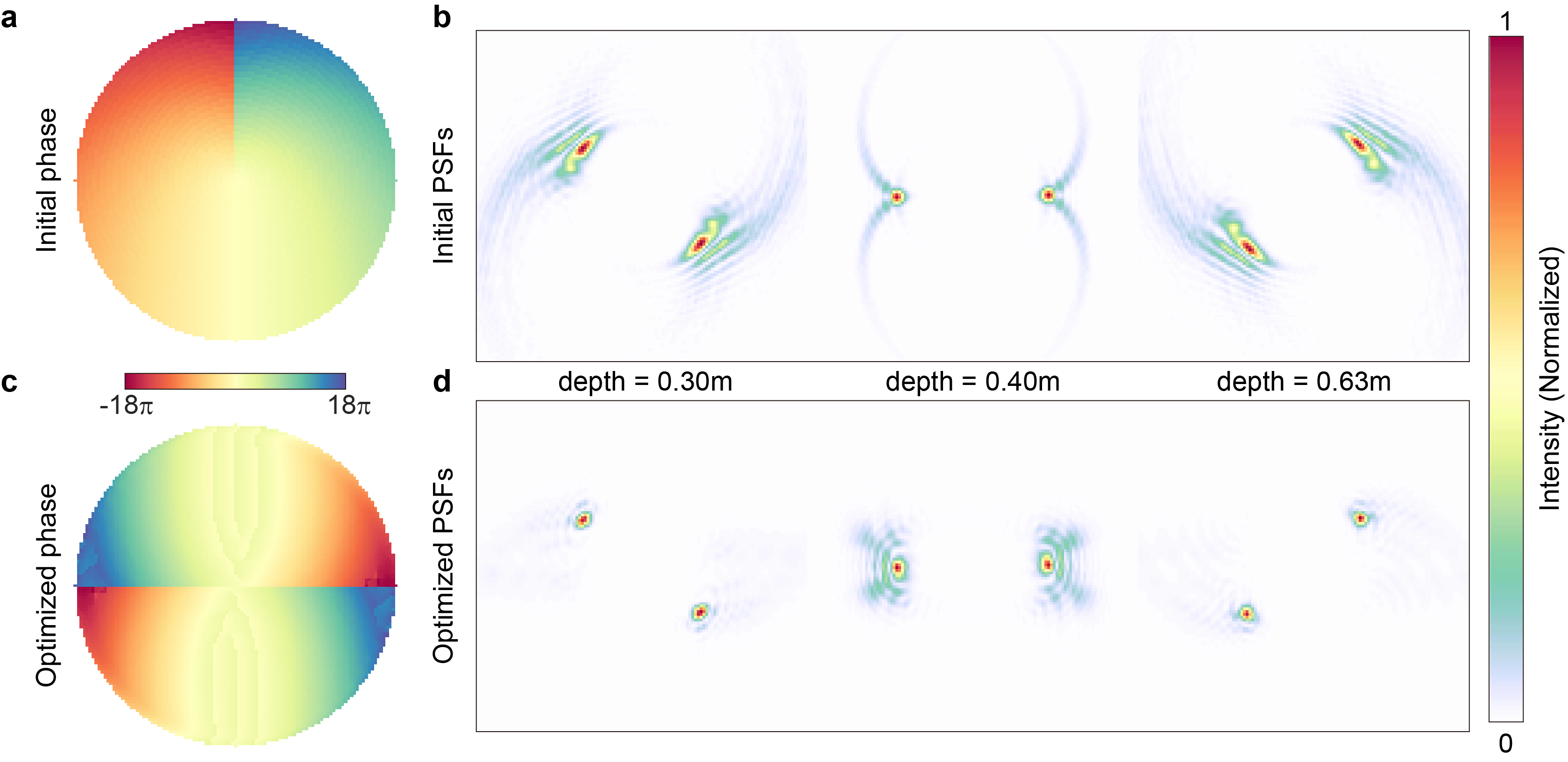
Supplementary Fig. S6** **| Initial and optimized phase mask and PSFs. a.** Initial Fresnel zone phase mask. **b.** Initial double-helix PSFs of 2D cameras. **c.** Optimized phase mask. **d.** Optimized double-helix PSFs of 2D cameras.


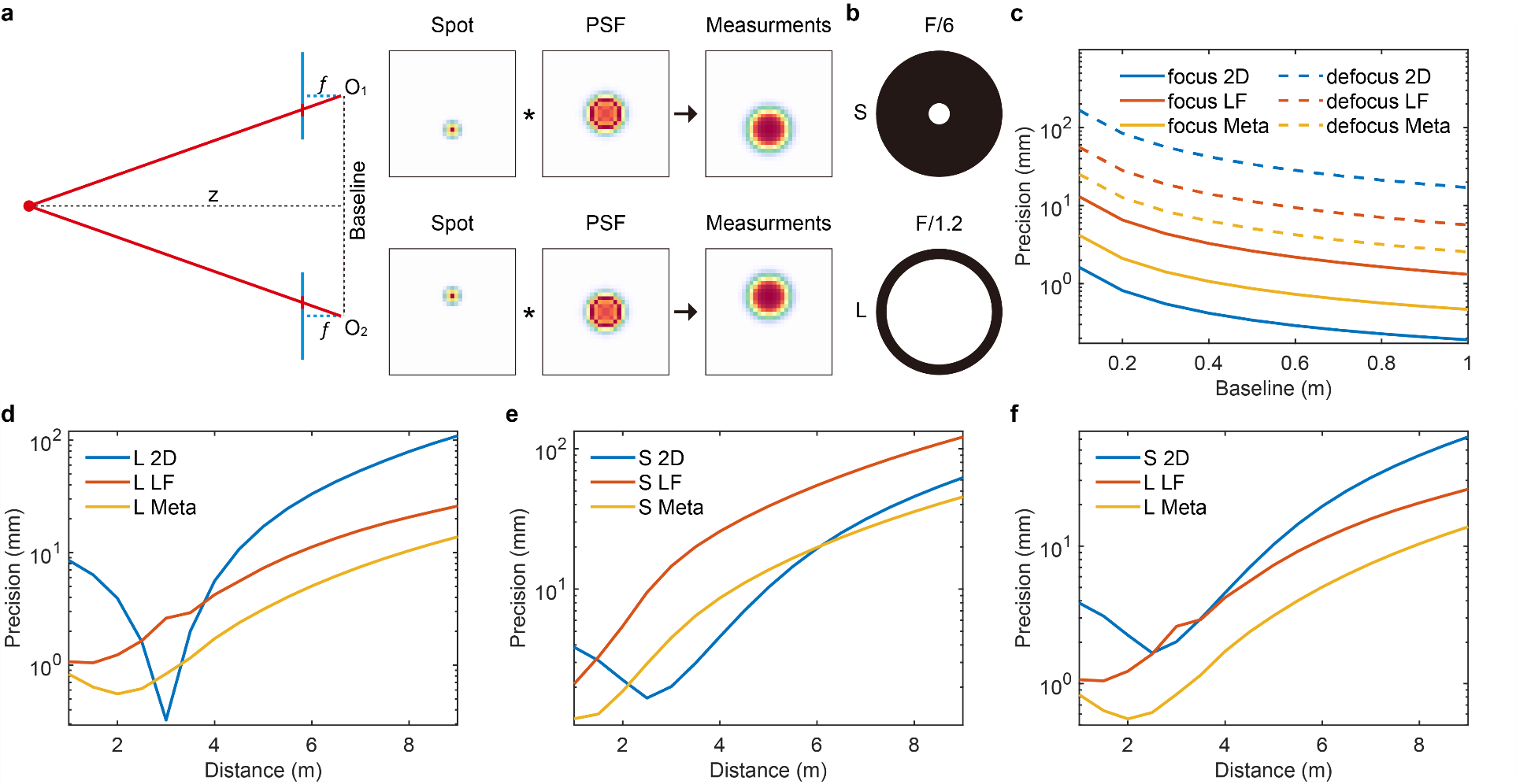
**Supplementary Fig. S7** **| Depth sensing precision of binocular stereo vision system using different cameras.** The focus length of the main lens is 25mm for all cameras. “L” represents a large aperture (f/1.2), and “S” represents a small aperture (f/6). The focus distance is 3m for all cameras. The signal-background ratio is 20dB and the standard deviation of the Gaussian spot is 1.85um, equal to the pixel size of the sensor. **a.** Forward model of a binocular stereo vision system. Assuming a Gaussian spot projects onto a binocular system with a horizontal baseline, we calculate the positions of the spots on the two cameras using geometric optics. We then convolve the spots with the point spread functions (PSF) of the cameras to obtain the measurements of the stereo vision system. **b.** Visualization of small aperture and large aperture. **c.** The curve of depth sensing precision at the focus (3m) and defocus (6m) positions versus varying baseline. The aperture is “L”. **d-f.** Depth sensing precision at various distances under different apertures. The baseline is 0.1m.

**
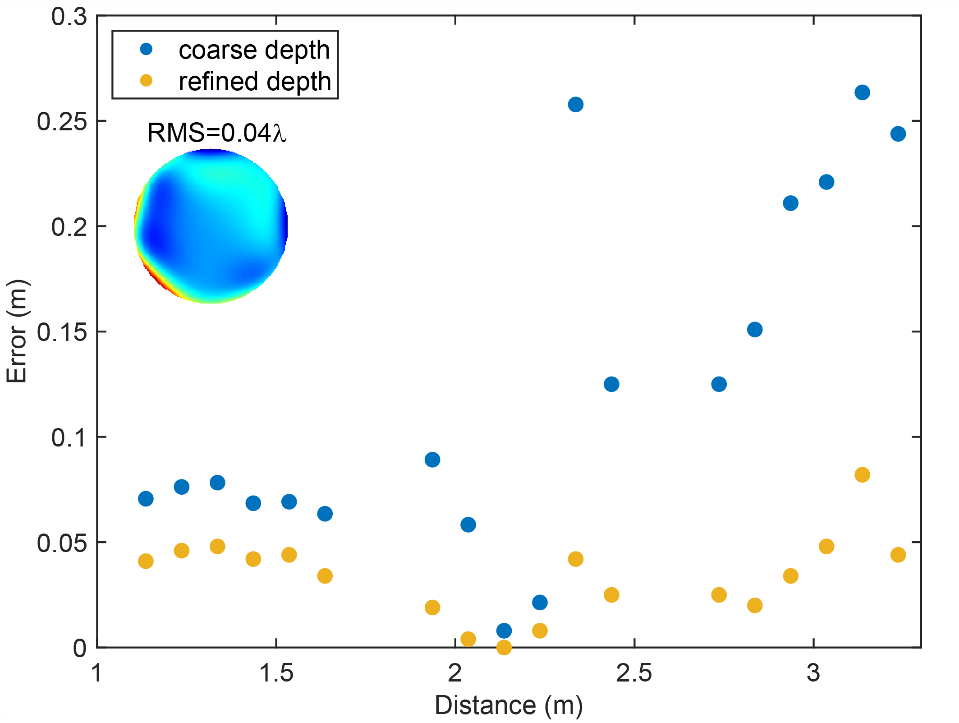
****Supplementary Fig. S8 | Estimation error of coarse depth and refined depth.** The estimated system aberration by the meta-imaging camera is displayed in the upper left corner. The refined depth obtained after 3D PSF fitting is more accurate than the coarse depth estimated from defocus aberration reconstructed from deconvolution.


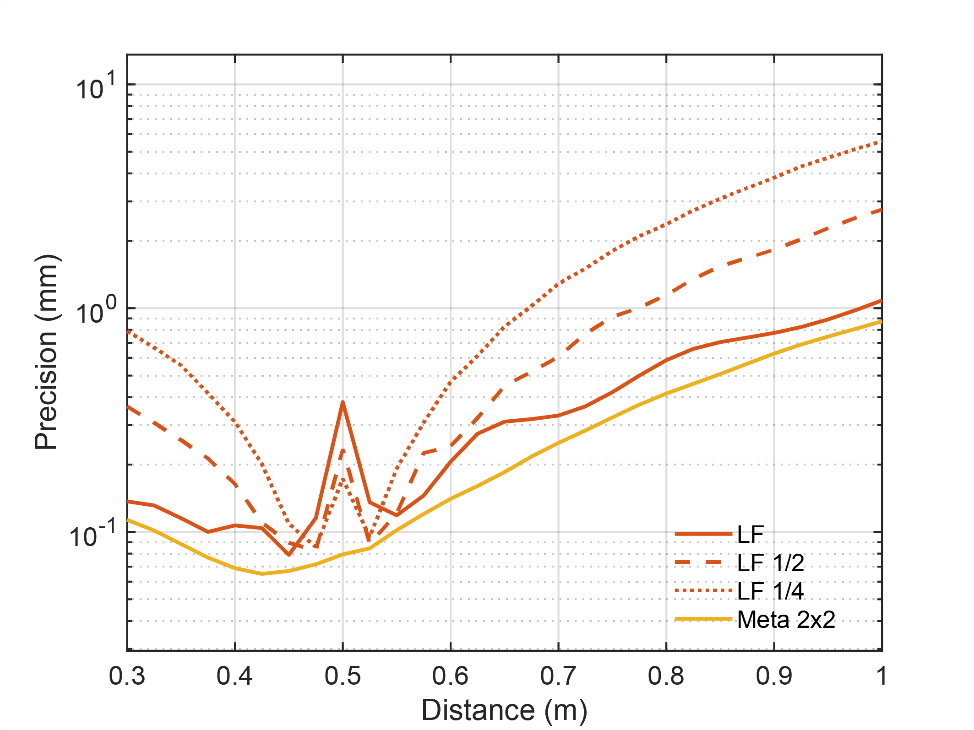
**Supplementary Fig. S9** **| The curve of depth sensing precision of 2x2 meta-imaging cameras and light-field cameras with different pixel sizes.** The focus length of the main lens is 25mm for all cameras and the f-number is 6. “Meta 2x2” represents a large pixel size (1.85um) and scanning 2x2. “LF 1/2” represents a smaller pixel size (0.925um) and smaller MLA pitch (7.4um). “LF 1/4” represents the smallest pixel size (0.4625um) and smallest MLA pitch (3.7um). The focus distance is 0.5m for all cameras. Performance improvement in depth sensing achieved through the higher spatial resolution brought by denser camera sensors is degraded by the corresponding decrease in angular resolution and increased noise level.

## Supplementary Notes

### Supplementary Note S1 | Detailed propagation formula

As shown in Fig.1 The propagation process can be mathematically modeled by Fourier optics. The derivations of each propagation process are as follows. For the first process, the point source will propagate in the form of a spherical wave. Thus, the complex field in front of the main lens can be written as

$$\begin{aligned} U_{1}\left( x,y \right)=A\frac{e^{jkr}}{r},\quad r=\sqrt{x^{2}+y^{2}+z^{2}}\#\left( SEQ eq 1 \right) \end{aligned}$$

Where $k$ is the wave number. Then the complex field passes through the main lens and the modulation process is given by

$$\begin{aligned} U_{1}^{'}\left( x,y \right)=U_{1}\left( x,y \right)\cdot t_{ML}\left( x,y \right)\#\left( SEQ eq 2 \right) \end{aligned}$$

$$\begin{aligned} t_{ML}\left( x,y \right)=P_{ML}\left( x,y \right)\exp\left[ -j\frac{k}{2f}\left( x^{2}+y^{2} \right) \right]\#\left( SEQ eq 3 \right) \end{aligned}$$

$$\begin{aligned} P_{ML}\left( x,y \right)=\left\{ \begin{aligned} 1, x^{2}+y^{2}\leq R_{1}^{2} \\ 0, otherwise \end{aligned} \right.\#\left( SEQ eq 4 \right) \end{aligned}$$

Where $R_{1}$ is the radius of the main lens and $f$ is the focus length of the main lens. After modulation of the main lens, the complex field propagates from the rear surface of the main lens to the front of MLA. According to Fresnel diffraction theory, the process can be expressed as

$$\begin{aligned} U_{2}\left( x,y \right)=U_{1}^{'}\left( x,y \right)*h_{1}\left( x,y \right)\#\left( SEQ eq 5 \right) \end{aligned}$$

$$\begin{aligned} h_{1}\left( x,y \right)=\frac{e^{jkz_{0}}}{j\lambda z_{0}}\exp\left[ \frac{jk}{2z_{0}}\left( x^{2}+y^{2} \right) \right]\#\left( SEQ eq 6 \right) \end{aligned}$$

Where $\lambda$ is the wavelength, $*$ denotes convolution, $z_{0}$ is the distance between the main lens and microlens array, $U_{2}$ represents the complex field on the front face of MLA, which is the PSF of the 2D camera as well. Denotes the center coordinate of each pixel as$(x_{i},y_{i}),$ and the discrete PSF can be written as

$$\begin{aligned} 2D\left( i,j \right)=\int_{x_{i}-\Delta}^{x_{i}+\Delta} \int_{y_{j}-\Delta}^{y_{j}+\Delta} U_{2}\left( x,y \right)dydx\#\left( SEQ eq 7 \right) \end{aligned}$$

Then the complex field passes through MLA and the modulation process can be directly derived from Eq.$\left( 2 \right)$ by substituting the $U_{1}^{'}$ with $U_{2}^{'}$, $U_{1}$ with $U_{2}$, and $t_{ML}$ with $t_{MLA}$. Denoting center coordinate of each microlens as $\left( u_{i},v_{j} \right)$, $t_{MLA}$ can be given by

$$\begin{aligned} t_{MLA}\left( x,y \right)=\sum_{i,j} P_{microlens}\left( x,y;u_{i},v_{j} \right)\exp\left[ -j\frac{k}{2f}\left( \left( x-u_{i} \right)^{2}+\left( y-v_{j} \right)^{2} \right) \right]\#\left( SEQ eq 8 \right) \end{aligned}$$

$$\begin{aligned} P_{microlens}\left( x,y;u_{i},v_{j} \right)=\left\{ \begin{aligned} 1, \left( x-u_{i} \right)^{2}+\left( y-v_{i} \right)^{2}\leq R_{2}^{2} \\ 0, otherwise \end{aligned} \right.\#\left( SEQ eq 9 \right) \end{aligned}$$

After phase modulation of MLA, the complex field $U_{2}$ propagates from the MLA to the sensor plane. The process can be modeled using Eq.$\left( 5 \right)$ by substituting $U_{2}$ with $U_{3}$, $U_{1}^{'}$ with $U_{2}^{'}$, and $h_{1}$ with $h_{2}$. To obtain $h_{2}$, we can use Eq.$\left( 6 \right)$ by substituting $z_{0}$ with $f_{mla}$, where $f_{mla}$, is the microlens focus length. By using these equations, we can derive the PSF of the light field camera, which also serves as the PSF of the meta-imaging camera when the scan number is 1. The discrete PSF of the light-field camera can then be represented as

$$\begin{aligned} LF\left( i,j,m,n \right)=\int_{u_{i}+x_{m}+\Delta}^{u_{i}+x_{m}+\Delta} \int_{v_{j}+y_{n}-\Delta}^{v_{j}+y_{n}+\Delta} U_{3}\left( x,y \right)dydx\#\left( SEQ eq 10 \right) \end{aligned}$$

Where, $\left( x_{m},y_{n} \right)$ denotes the center coordinate of each pixel relative to $\left( u_{i},v_{j} \right)$ and $\Delta$ denotes half of the pixel size. Here $\left( x_{m},y_{n} \right)$ represents angular coordinates and $\left( u_{i},v_{j} \right)$ represents spatial coordinates as well.

For the meta-imaging camera, we can obtain $N\times N$ complex fields by shifting $U_{2}$ and propagating shifted $U_{2}$ through Eq.$\left( 5 \right)$ $\left( 8 \right)$ $\left( 9 \right)$ during $N\times N$ scanning. Then we can calculate corresponding measurements $LF_{\left( 1,1 \right)},LF_{\left( 1,2 \right)},\cdots,LF_{\left( N,N \right)}$ by substituting $U_{3}\left( x,y \right)$ in Eq.$\left( 10 \right)$. Then the discrete PSF of the meta-imaging camera can be written by performing pixel realignment as follows

$$\begin{aligned} Meta\left( i,j,m,n \right)={LF}_{\left( N-i\%N,N-j\%N \right)}\left( i//N,j//N,m,n \right)\#\left( SEQ eq 11 \right) \end{aligned}$$

Where $\%$ denotes modulus and $//$ denotes exact division. Note that the parity of the scan number should match that of the angle number.

### Supplementary Note S2 | Baseline of light-field cameras

The baseline between views in the meta-imaging sensor is directly proportional to the aperture of the main lens^1^. Given aperture size $D$, focus length $f$, angular resolution $N$, and pixel size $\Delta p$. The vertical or horizontal baseline and disparity between views that are vertically or horizontally k units apart from each other can be given by

$$\begin{aligned} B=\frac{k}{N}D,\quad k=1,2,\cdots,N\#\left( SEQ eq 12 \right) \end{aligned}$$

$$\begin{aligned} disp=\frac{Bf}{\Delta p}(\frac{1}{z}-\frac{1}{z_{0}})\#\left( SEQ eq 13 \right) \end{aligned}$$

### Supplementary Note S3 | Principle of double-helix phase mask design

To generate a rotating double-helix PSF at wavelength λ = 525 nm, we assume the placement of a phase mask at the entrance pupil of the imaging system, and initialize the transmission phase of the phase mask with Fresnel zones, which can be mathematically expressed as^2^,

$$\begin{aligned} \psi_{r}\left( r,\theta\right)=\left\{ \theta\left( 2l-1 \right)\left| \left( \frac{l-1}{L} \right)^{\varepsilon}\leq r\leq\left( \frac{l}{L} \right)^{\varepsilon} \right.,l=1,\ldots,L \right\}\#\left( SEQ eq 14 \right) \end{aligned}$$

Where $r$ is the normalized radial coordinate and $\theta$ is the azimuth angle in the entrance pupil plane. Here, $[L, \varepsilon]$ are design parameters. An iterative Fourier transform algorithm is then utilized to maximize the energy in the main lobe of the rotating PSF within the specified rotation range. In each iteration, a Gaussian weight is applied to the helix to enhance the energy of the main lobe. The phase of the inverse Fourier transform of the Gaussian-weighted helix is then used as the new phase mask. For the phase mask we used in Fig. 5, we set $\left[ L, \varepsilon\right]=[24, 1.0]$ and the iterative optimization further improves the peak intensity of the main lobe of each helix by 65% on average.

### Supplementary Note S4 | The importance of scanning mechanism for depth sensing performance improvement

The performance of the meta-imaging camera scanning 8x8 times within a microlens area (8x8 meta-imaging camera) is slightly better than that of the meta-imaging camera scanning 2x2 times within a microlens area (2x2 meta-imaging camera). However, the performance improvement from increasing the number of scans from 2x2 to 8x8, reflected in the reduction of RMSE from 13mm to 9mm, is less pronounced compared to the improvement achieved by replacing a light field camera with a 2x2 meta-imaging camera, which reduces RMSE from 32mm to 13mm (Supplementary Fig. S5). Given that the cost of 8x8 scanning is 16 times higher than that of 2x2 scanning, we describe this performance enhancement as "slight".

However, this does not necessarily imply that the traditional light-field camera with a 4x denser camera sensor would achieve comparable performance to the meta-imaging camera. The scanning mechanism achieves performance improvement by maintaining the physical size of both microlenses and pixels while moving the microlens to improve spatial resolution, thus overcoming the inherent trade-off between spatial and angular resolution. This mechanism cannot simply be replaced by a denser camera sensor due to this trade-off and the increased noise levels associated with smaller pixel sizes. Supplementary Fig. S9 illustrates that performance improvement in depth sensing achieved through the higher spatial resolution brought by denser camera sensors is degraded by the corresponding decrease in angular resolution.

## References

1. Hahne, C. et al. Baseline and triangulation geometry in a standard plenoptic camera. *International Journal of Computer Vision* **126**, 21–35 (2018).

2. Berlich, R. & Stallinga, S. High-order-helix point spread functions for monocular three-dimensional imaging with superior aberration robustness. *Optics Express* **26**, 4873–4891 (2018).
